# The EPA guidance publications on neurocognition in schizophrenia

**DOI:** 10.1192/j.eurpsy.2025.209

**Published:** 2025-08-26

**Authors:** A. Vita

**Affiliations:** Experimental and Clinical Sciences, University of Brescia, Brescia, Italy

## Abstract

**Abstract: Introduction:**

Although cognitive impairment is a core symptom of schizophrenia related to poor outcome in different functional domains, it still remains a major therapeutic challenge. To date, cognition is still poorly assessed in both research and clinical settings and no comprehensive treatment guidelines for assessment and treatment of cognitive impairment in schizophrenia are implemented.

**Objectives:**

The aim of the European Psychiatric Association (EPA) guidance paper was to provide a comprehensive meta-review of the current available evidence for the assessment of cognitive functions in schizophrenia both in research settings and in real-world clinical practice and for treatment of cognitive impairment, structured into three sections: pharmacological treatment, psychosocial interventions, and somatic treatments.

**Results:**

Based on the reviewed evidence, the EPA guidance recommends a comprehensive and systematic assessment of neurocognitive and social cognitive domains in schizophrenia, in all phases of the disorder, as well as in subjects at risk to develop psychosis. It is recommended not only the use of observer reports, but also of self-reports and interview-based cognitive assessment tools. As for treatment, the EPA guidance recommends an appropriate pharmacological management as a fundamental starting point in the treatment of cognitive symptoms in schizophrenia. Among psychosocial interventions, cognitive remediation and physical exercise, and some variables have been confirmed as core elements for cognitive remediation effectiveness.

**Conclusion:**

The dissemination of the EPA guidance papers may promote the development of shared guidelines concerning the assessment and treatment of cognitive functions in schizophrenia, with the purpose to improve the quality of care and to achieve clinical recovery in this population.

**Disclosure of Interest:**

None Declared

